# Gene and Cell Therapy in Regenerative Medicine—Second Edition

**DOI:** 10.3390/cells15060560

**Published:** 2026-03-20

**Authors:** Albert A. Rizvanov, Ayşegül Doğan

**Affiliations:** 1 Institute of Fundamental Medicine and Biology, Kazan (Volga Region) Federal University, Kazan 420008, Russia; 2 Division of Medical and Biological Sciences, Tatarstan Academy of Sciences, Kazan 420111, Russia; 3 Faculty of Engineering and Natural Sciences, Genetics and Bioengineering Department, Yeditepe University, İstanbul 34755, Türkiye

## 1. Introduction

Gene and cell therapies have expanded the toolbox of regenerative medicine by enabling (i) targeted modulation of tissue repair programs, (ii) replacement of damaged or missing cell populations, and (iii) durable correction of molecular defects that prevent regeneration. At the same time, clinical translation increasingly depends on practical questions—delivery, manufacturing consistency, safety monitoring, and appropriate endpoints that reflect functional recovery rather than surrogate biomarker changes alone.

This Special Issue, “Gene and Cell Therapy in Regenerative Medicine—Second Edition”, brings together eight open-access contributions spanning ocular regenerative approaches, inflammation-aware cartilage repair, topical nucleic acid therapeutics for chronic wounds, secretome-based cytoprotection in kidney injury models, next-generation lentiviral vector design for β-hemoglobinopathies, patient-specific induced pluripotent stem cell (iPSC) disease modeling, and pathway-focused perspectives on neuroinflammation in spinal cord injury. Collectively, these articles emphasize that regenerative outcomes are rarely determined by a single component; rather, they emerge from the interaction between therapeutic payloads, recipient microenvironments, and immune/inflammatory context.

## 2. Field Advances and Trends in 2025–2026

Several developments from 2025 to 2026 are particularly relevant for regenerative medicine at the interface of gene delivery, cell engineering, and translational feasibility.

### 2.1. Precision Genome Editing Moves Beyond “Cut-and-Repair”

While CRISPR-Cas nucleases remain foundational, 2025 provided early clinical signals that “search-and-replace” editing paradigms can be implemented in patients. Prime editing was reported in a first-in-human setting for a rare immune disorder, illustrating the translational potential of editing strategies that can correct pathogenic variants without requiring double-strand breaks. Such approaches are conceptually important for regenerative medicine because they may reduce genotoxicity risks and expand the range of correctable variants in autologous cell products [1].

### 2.2. N-of-1 Gene Editing as an Actionable Workflow

A second, practical shift is the emergence of rapid development pathways for individualized therapies. In 2025, patient-specific in vivo base editing for a severe ultrarare metabolic disorder was reported in a peer-reviewed clinical format. Beyond its immediate clinical relevance, this case is a proof-of-principle for an accelerated design-to-dose pipeline (variant interpretation → editor selection → formulation and manufacturing → regulatory review → dosing) that may become increasingly relevant for monogenic conditions with early, irreversible tissue damage where classical drug development timelines are incompatible with disease progression [2].

### 2.3. Sensory Organ Gene Therapy Reaches Clearer Functional Readout

Regeneration-oriented gene therapy has also benefited from anatomical compartments in which local delivery and direct functional outcomes are measurable. In 2025, clinical results for AAV-based gene therapy targeting OTOF-related inherited deafness were reported, including improvements consistent with restoration of auditory function in treated individuals. Sensory tissues (eye, inner ear) remain prominent for regenerative strategies because they allow localized administration, potentially lower systemic exposure, and quantifiable functional endpoints [3].

### 2.4. Delivery Diversification: Engineered Biological Carriers and Cell-Free Modalities

Delivery remains a central constraint for both nucleic-acid therapeutics and intracellular protein/RNP payloads. In 2025, engineered extracellular vesicles (EVs) were reported as a platform for efficient intracellular delivery of multimodal therapeutics, including genome editors, highlighting sustained interest in biological carriers that may complement (rather than replace) viral vectors and synthetic nanoparticles. In parallel, cell-free products such as conditioned media, secretomes, and EV fractions are gaining traction in regenerative medicine as mechanisms to capture beneficial paracrine effects while simplifying storage, dosing, and safety management compared with living cells [4].

### 2.5. Epigenetic Modulation and Partial Reprogramming Enter Early Clinical Positioning

An additional direction that gained visibility by 2026 is the attempt to restore tissue function by controlled epigenetic state modulation (often referred to as partial reprogramming). Early-stage clinical positioning in ocular indications has been publicly communicated, reflecting the view that some degenerative conditions may be approached by resetting cellular programs rather than replacing cells. For regenerative medicine, this direction is notable because it reframes “repair” as a programmable state transition—while also raising questions about dose control, durability, and long-term surveillance [5].

These emerging directions also define several practical questions currently shaping research in regenerative medicine: how to improve therapeutic cell function in hostile microenvironments, how to deliver transient genetic signals to guide tissue repair, how to design vectors that combine multiple therapeutic mechanisms, and how to better model disease mechanisms for therapy development.

The articles collected in this Special Issue address these questions from different angles. To highlight their conceptual connections with the broader developments described above, the contributions can be grouped into several thematic areas reflecting key challenges of gene and cell therapy in regenerative medicine.

## 3. Overview of Published Articles in This Special Issue

The eight contributions included in this Special Issue address several key challenges currently shaping gene and cell therapy in regenerative medicine. These studies can be grouped into several thematic areas reflecting how different technological approaches aim to solve specific translational problems in the field.

### 3.1. Ophthalmological Regeneration and Ocular Cell Protection

One important direction in regenerative medicine concerns sensory tissues, where localized delivery and measurable functional outcomes make them attractive targets for gene and cell therapy. In this context, the review by Santa Cruz-Pavlovich et al. provides a structured overview of regenerative medicine strategies in ophthalmology, spanning cell therapies, exosomes, scaffolds, in vivo reprogramming, organoids, and interspecies chimerism and summarizes the clinical trial landscape across a range of ocular diseases. The breadth of approaches covered underscores the heterogeneity of ocular pathologies and the need to match modality selection to tissue compartment, disease mechanism, and feasible delivery route [6]. Together, these studies illustrate how both cell-based and molecular approaches can be adapted to address oxidative stress and tissue degeneration in ocular diseases.

Complementing this systems-level perspective, Lee et al. investigated a practical “potency enhancement” strategy for mesenchymal stromal cell therapy in ocular settings. In an in vitro injury model using human retinal pigment epithelial cells, the authors reported that priming placenta-derived MSCs with Achyranthis radix extract increased proliferative capacity, delayed senescence, and enhanced antioxidant-related and regeneration-associated gene expression, supporting the concept that controlled preconditioning may amplify the functional output of MSC products in oxidative stress-rich environments [7].

### 3.2. Musculoskeletal Repair Under Inflammatory Constraints

Another important question in regenerative medicine concerns how inflammatory microenvironments influence therapeutic outcomes. Obermeyer et al. compared minced cartilage implantation (MCI) with autologous chondrocyte transplantation (ACT)-like conditions in a standardized TNFα-driven in vitro inflammation model.

Inflammation is a key determinant of cartilage repair outcomes, particularly in osteoarthritic contexts. Obermeyer et al. compared minced cartilage implantation (MCI) with autologous chondrocyte transplantation (ACT)-like conditions in a standardized TNFα-driven in vitro inflammation model. Their data indicate that MCI constructs were less susceptible to TNFα-associated impairment than passaged chondrocytes, including reduced IL-6 release and smaller shifts in inflammation markers. This type of work is relevant for translating cartilage repair strategies into real-world inflammatory microenvironments rather than idealized conditions [8]. These findings highlight the importance of evaluating regenerative strategies under disease-relevant inflammatory conditions.

### 3.3. Local Nucleic-Acid Therapeutics for Chronic Wound Repair

Chronic diabetic wounds remain a clinically demanding indication in which angiogenesis and tissue remodeling are limiting steps. Transient gene delivery represents another emerging strategy to guide tissue repair without permanent genome modification. In this context, Thang et al. evaluated topical delivery of modified mRNAs encoding VEGF-A and FGF1 and reported improved vascular sprouting ex vivo and accelerated wound closure with increased neovascularization in diabetic mice. Their transcriptional profiling supports that combined growth-factor expression can reshape early wound programs. The study illustrates an emerging theme in regenerative medicine: the use of transient, local nucleic acid-encoded signals to steer repair without requiring permanent genomic change [9].

### 3.4. Paracrine and Cell-Free Interventions in Kidney Injury Models

In addition to direct gene or cell therapies, increasing attention is being given to paracrine and cell-free therapeutic approaches. The work by Rendra et al. addresses cisplatin-induced kidney injury through the lens of cell–immune crosstalk. Using adipose stromal cell-derived conditioned medium, the authors reported protection of proximal tubule epithelial cells via reduced oxidative stress and apoptosis and immunomodulatory effects on macrophages consistent with an M2-like shift and increased phagocytosis. Importantly, the study design explicitly evaluates the bidirectional interaction between epithelial and immune compartments, aligning with the broader need to treat injury–inflammation feedback loops rather than targeting only one component of pathology [10].

### 3.5. Vector Design and Combinatorial Strategies for β-Hemoglobinopathies

Genetic diseases requiring durable correction continue to drive innovations in vector engineering. Gene addition remains a key approach for inherited disorders where durable correction is required. Fleischauer et al. evaluated mono- and bifunctional GLOBE-based lentiviral vectors for β-thalassemia therapy, focusing on strategies to increase β-like globin expression without escalating vector dose. The authors describe a GLOBE-derived vector incorporating an HBB transcription terminator element and examine mutation-specific RNA interference designs (including miRNA-embedded shRNAs) alone and in combination with βAS3-globin expression. Such combinatorial constructs are relevant beyond hemoglobinopathies because they exemplify multi-layer engineering (expression optimization + allele-specific suppression) within a single vector architecture [11].

### 3.6. Patient-Specific iPSC Models and Ultrastructural Phenotyping in Rare Disease

Alongside therapeutic development, disease modeling using induced pluripotent stem cells has become an important enabling platform for regenerative medicine. Disease modeling remains an enabling technology for regenerative medicine, particularly for conditions in which mechanistic insight and screening platforms are prerequisites for therapy development. Shnaider et al. generated iPSCs from patients with Cohen syndrome and differentiated them into neural stem cells and neurons, followed by transmission electron microscopy-based characterization. The authors report organelle-level abnormalities (Golgi, endoplasmic reticulum, mitochondria) and accumulation of autophagosomes, linking VPS13B dysfunction to cellular phenotypes that resemble broader neurodegenerative patterns. Such fine-grained phenotyping can support target discovery, candidate selection, and assessment of intervention reversibility in vitro [12].

### 3.7. Neuroinflammation Signaling as a Lever for CNS Repair

For central nervous system injuries, modulation of inflammatory signaling pathways remains a critical therapeutic target. Finally, the review by Ageeva et al. focuses on NF-κB and JAK/STAT pathways as regulators of neuroinflammation and astrocyte modulation in spinal cord injury. By synthesizing evidence on pathway interactions, astroglial responses, and glial scar dynamics, the authors outline why pathway-targeted interventions (including gene and cell therapy strategies designed to tune inflammatory signaling) remain central for improving functional recovery after CNS injury [13].

## 4. Conclusions and Outlook

Taken together, the studies included in this Special Issue illustrate how different technological approaches address key biological and translational challenges of regenerative medicine. Across diverse indications, several common principles emerge from these contributions. First, regenerative performance is strongly shaped by inflammatory and oxidative stress contexts; therefore, therapies must be tested and optimized under relevant injury-mimicking conditions. Second, there is a continued shift from “single-component” interventions to modular, combinatorial designs—whether by priming cells, combining growth-factor signals, or integrating gene addition with mutation-specific suppression. Third, translational readiness increasingly depends on manufacturability and controllability, motivating interest in cell-free products, transient nucleic-acid therapeutics, and delivery carriers that broaden feasible payloads.

Looking forward, 2025–2026 developments in prime editing, individualized in vivo gene editing, engineered EV carriers, and early clinical positioning of epigenetic modulation suggest that regenerative medicine will keep expanding its mechanistic range—from replacement and repair toward programmable restoration of cellular state. The next stage will require careful alignment between technological capability and clinical reality: robust product characterization, clinically meaningful endpoints, and long-term surveillance frameworks appropriate for durable or state-altering interventions.
